# Short-Course, Low-Dose Metoclopramide as Bridge Therapy for Dysautonomia-Associated Gastrointestinal Dysmotility in Adolescents: A Case Series

**DOI:** 10.3390/children13070960

**Published:** 2026-07-21

**Authors:** Alexandra Wilder, Cynthia Morris, Dhiren Patel, Aniruddh Setya

**Affiliations:** 1 Department of Pediatrics, Saint Louis University School of Medicine, St. Louis, MO 63104, USAcynthia.morris@slucare.ssmhealth.com (C.M.); dhiren.patel@slucare.ssmhealth.com (D.P.); 2 Division of Pediatric Neurology, SSM Health Cardinal Glennon Children’s Hospital, St. Louis, MO 63104, USA; 3 Division of Pediatric Gastroenterology, Hepatology and Nutrition, Cardinal Glennon Children’s Hospital, St. Louis, MO 63104, USA

**Keywords:** dysautonomia, postural orthostatic tachycardia syndrome, POTS, metoclopramide, gastrointestinal dysmotility, functional dyspepsia, prokinetic, pediatric, bridge therapy, tardive dyskinesia

## Abstract

**Highlights:**

**What are the main findings?**

- Short-course, low-dose metoclopramide was associated with meaningful improvement in nausea and gastrointestinal symptoms in adolescents with dysautonomia-associated GI dysmotility.
- No extrapyramidal symptoms or serious adverse effects were observed across all cases, with cumulative doses well below established risk thresholds for tardive dyskinesia.

**What are the implications of the main findings?**

- Metoclopramide may serve as a safe, time-limited “bridge therapy” to enable nutritional rehabilitation and multidisciplinary treatment engagement in this population.
- The perceived risk of tardive dyskinesia with short-term, low-dose use in adolescents may warrant further investigation in this specific population; prospective studies are needed to better define safety and efficacy.

**Abstract:**

Background: Dysautonomia, including postural orthostatic tachycardia syndrome (POTS) and related orthostatic disorders, is frequently associated with debilitating gastrointestinal (GI) symptoms in adolescents, including chronic nausea, early satiety, postprandial fullness, and functional dysmotility. Despite the significant disease burden, pharmacological options for GI dysmotility in this population remain poorly studied. To our knowledge, no prior case series has described metoclopramide as a targeted prokinetic bridge therapy specifically in adolescents with dysautonomia-associated GI dysmotility symptoms. Case Series Summary: We describe three adolescent females (ages 13, 16, and 16 years) with specialist-confirmed dysautonomia and refractory GI symptoms who were treated with a standardized short-course, low-dose metoclopramide bridge protocol (5 mg three times daily, tapered over approximately 12 weeks). All patients received concurrent multidisciplinary management including dietary modification, neuromodulators, integrative therapies, and behavioral support. All three demonstrated subjective improvement in nausea and GI symptoms, enabling engagement with broader rehabilitative and nutritional interventions. No extrapyramidal symptoms or serious adverse effects were observed. Cumulative metoclopramide doses across all three cases ranged from approximately 770 to 1050 mg—well below the threshold associated with tardive dyskinesia risk in contemporary real-world data. Conclusions: Short-course, low-dose metoclopramide, administered as part of a structured multidisciplinary protocol with explicit safety counseling and planned taper, may serve as a feasible bridge therapy for adolescents with dysautonomia-associated GI dysmotility symptoms. These observations are hypothesis-generating and should be interpreted with caution given the small sample size, concurrent multidisciplinary interventions, and absence of standardized outcome instruments. They support the need for prospective investigation of short-course, weight-dosed metoclopramide use in this specific population.

## 1. Introduction

Dysautonomia encompasses a spectrum of disorders characterized by dysfunction of the autonomic nervous system (ANS), with postural orthostatic tachycardia syndrome (POTS) representing the most commonly identified subtype in adolescents [1,2]. These conditions occur predominantly in females and are increasingly recognized in the pediatric population [2]. A substantial gastrointestinal (GI) burden has been documented: pooled data from six studies (N = 352 patients) reported a prevalence of approximately 69% for nausea and abdominal pain—the most frequent noncardiovascular symptoms in POTS [3]. A larger and more recent systematic review and meta-analysis (19 studies, *n* = 8268) corroborates this burden, reporting at least one GI symptom in 57.9% of POTS patients, with nausea the most prevalent complaint (70.1%) [4]. Reported GI symptoms include chronic nausea, early satiety, bloating, postprandial pain, and functional dyspepsia [3,5]. Among pediatric cohorts with POTS or orthostatic intolerance, approximately 33–42% have been shown to have abnormal gastric emptying on objective testing [6]. The pathophysiology is multifactorial, encompassing autonomic dysregulation of gastric motility and emptying, visceral hypersensitivity, impaired splanchnic perfusion, and bidirectional gut–brain axis dysfunction [6,7].

The GI manifestations of dysautonomia are frequently debilitating. Impaired oral intake, nutritional compromise, and functional disability can prevent adolescents from participating in school, physical activity, and social life—compounding the psychosocial burden already associated with chronic dysautonomia [7,8]. Treatment remains challenging. Multidisciplinary approaches that integrate dietary modification, behavioral therapy, physical reconditioning, complementary interventions, and judicious pharmacotherapy are increasingly recognized as essential to recovery [6,7]. However, initiating non-pharmacological strategies is often impractical when patients are debilitated by severe nausea and severely restricted oral intake. A pharmacological strategy capable of providing rapid, time-limited symptom relief—a ‘bridge’—while enabling engagement with the broader therapeutic program represents an important unmet clinical need.

Metoclopramide, a dopamine D2 receptor antagonist with combined prokinetic and antiemetic properties, is the only FDA-approved oral prokinetic available in the United States [9,10,11]. Its dual peripheral mechanism—accelerating gastric emptying and enhancing antroduodenal coordination—and central antiemetic action at the chemoreceptor trigger zone make it mechanistically well-suited to the dysmotility and nausea profile of dysautonomia [9,12]. However, the FDA’s 2009 black box warning regarding tardive dyskinesia (TD) risk, and the subsequent labeling stating that metoclopramide tablets are ‘not recommended for use in pediatric patients,’ have substantially curtailed its use [13]. These restrictions were grounded principally in data from chronic, high-dose adult use; contemporary real-world evidence has provided additional context, suggesting that TD incidence in short-course use may be lower than historical estimates in selected populations [12,14]. Pediatric gastroparesis research more broadly remains limited: a systematic review identified only 12 randomized controlled trials in this population, all restricted to infants, underscoring the paucity of evidence guiding prokinetic use in children and adolescents [15].

To our knowledge, following a structured PubMed search using the terms “metoclopramide AND pediatric AND (dysautonomia OR POTS OR orthostatic intolerance)”, we identified no prior published case series specifically describing the use of metoclopramide as a targeted bridge therapy in adolescents with dysautonomia-associated GI dysmotility. We present three such cases, treated with a standardized short-course, low-dose protocol under careful clinical monitoring, with detailed documentation of dosing, safety, and outcomes. We additionally contextualize our findings within the evolving real-world safety literature and propose a practical monitoring framework for clinicians considering this approach.

## 2. Case Presentations

This case series is reported in accordance with the CARE (CAse REport) guidelines for case report reporting standards [16].

### 2.1. Case 1—13-Year-Old Female with Dysautonomia and Chronic Nausea

Patient 1 is a 13-year-old female with a 3–4-year history of persistent nausea, intermittent vomiting, and early satiety in the setting of specialist-confirmed dysautonomia. Tilt table testing (TTT) performed by her dysautonomia specialist neurologist demonstrated orthostatic hypotension with reactive tachycardia. The nausea was characteristically persistent throughout the day, with intermittent postprandial exacerbations, and was associated with reduced appetite, prolonged mealtimes, and habitual intake of small volumes. She additionally reported constipation, headaches, and dizziness consistent with her autonomic diagnosis.

Her diagnostic evaluation was comprehensive. Esophagogastroduodenoscopy (EGD) with biopsies performed at a tertiary center demonstrated normal mucosa throughout the esophagus, stomach, and duodenum without histopathologic abnormality. Fluoroscopic upper GI series, abdominal and appendix ultrasound, and brain MRI with and without contrast were all unremarkable. Laboratory investigations were within normal limits. No structural, inflammatory, or neoplastic explanation for her symptoms was identified. Prior pharmacological trials included cyproheptadine, ginger preparations, Iberogast, and amitriptyline 10 mg nightly—the last with only partial benefit.

At her initial GI visit, the patient weighed 51.9 kg and was assessed as having dysautonomia-associated GI dysmotility with a functional dyspepsia component. She met Rome V criteria for functional dyspepsia (postprandial distress syndrome subtype), with bothersome postprandial fullness and early satiation occurring on at least 3 days per week, present for more than 2 months [17]. Metoclopramide 5 mg three times daily before meals was initiated. At this dose and weight, the mg/kg/dose was 0.096 mg/kg—well below the 0.15 mg/kg/dose weight-based threshold. The FDA black box warning regarding the risk of acute dystonic reactions and tardive dyskinesia was explained in detail to the patient and her guardian. Diphenhydramine was prescribed as a rescue agent with written instructions to discontinue metoclopramide and administer diphenhydramine immediately if symptoms of dystonia or marked restlessness occurred. Concurrent interventions included amitriptyline 10 mg nightly (continued from prior), magnesium glycinate 240 mg daily, Iberogast, ginger capsules 500 mg twice daily, dietary modifications (small frequent low-fat meals, improved breakfast intake), hydration and salt expansion, and compression garment use.

At 8-week follow-up, the patient reported complete resolution of nausea and abdominal pain, with regular bowel movements 4–5 times weekly, increased meal volume, and expanding dietary variety. Her weight had increased by 1.4 kg. Metoclopramide was tapered to 5 mg twice daily and amitriptyline was increased to 20 mg nightly to optimize neuromodulation. The patient subsequently self-tapered and discontinued metoclopramide entirely without symptom recurrence. At final GI visit, approximately 5 months from initiation), she remained symptom-free off prokinetic therapy, with stable weight, normalized bowel habits, and improved dietary intake. She was discharged from GI follow-up. No extrapyramidal symptoms, movement disorders, or other adverse effects attributable to metoclopramide were observed at any visit. Neurological examination—performed at each visit by the co-author pediatric neurologist (C.M.) and encompassing all elements assessed by the Abnormal Involuntary Movement Scale (AIMS), including orofacial, extremity, trunk, and gait assessment—was normal throughout. Estimated total metoclopramide exposure was approximately 900–1000 mg.

### 2.2. Case 2—16-Year-Old Female with Dysautonomia, Nausea, and Constipation

Patient 2 is a 16-year-old female with a 2–3-year history of chronic nausea, vomiting, postprandial fullness, early satiety, and constipation in the setting of specialist-confirmed dysautonomia. TTT performed by her dysautonomia specialist neurologist confirmed orthostatic hypotension. She consistently reported an exaggerated awareness of gastric fullness after meals, often describing that she could “feel everything she ate,” indicative of prominent visceral hypersensitivity. Her medical history was significant for anxiety disorder, ADHD (confirmed on neuropsychological evaluation, and iron-deficiency anemia (resolved). Current medications at presentation included escitalopram 20 mg daily and methylphenidate 10 mg for ADHD.

Metoclopramide 5 mg three times daily had been initiated approximately 6 weeks prior to her initial GI visit by another provider, with the patient reporting it had ‘made a huge difference’ for her nausea. At her first GI visit she weighed 56.6 kg (mg/kg/dose: 0.088 mg/kg). She met Rome V criteria for functional dyspepsia with overlapping postprandial distress syndrome (postprandial fullness, early satiation) and epigastric pain syndrome features (episodic epigastric pain), as well as Rome V criteria for functional constipation [17]. The prescription was formalized, the black box warning regarding acute dystonic reactions and tardive dyskinesia was documented and explained, and diphenhydramine was prescribed as a rescue agent. A comprehensive laboratory panel was obtained, including complete blood count, comprehensive metabolic panel, C-reactive protein, TSH, IgA, tissue transglutaminase antibody, fecal calprotectin, ferritin, lipase, GGT, and vitamin D—all results were within normal limits, excluding celiac disease, systemic inflammation, hepatobiliary dysfunction, and endocrine pathology. Fecal calprotectin was 15 μg/g, excluding significant intestinal inflammation. Electrocardiogram and echocardiogram were reported normal. No endoscopy was indicated on clinical grounds.

Concurrent GI interventions included a proton pump inhibitor (1 mg/kg/day), hyoscyamine PRN for abdominal spasms, osmotic laxative (polyethylene glycol 3350), stimulant laxative (senna), magnesium oxide 800 mg, Iberogast, and dietary modifications (small frequent low-fat meals). The patient was seen at three further visits one month apart. Across this period, she demonstrated progressive symptomatic improvement: by the third visit she reported markedly reduced nausea, improved bowel frequency, and no abdominal pain. At this juncture, she executed a pragmatic de-escalation of metoclopramide to 5 mg only before a meal when she was going to eat, rather than maintaining scheduled TID dosing. At her final GI visit, metoclopramide was stopped given approximately 11 weeks of GI-supervised treatment and sustained symptom improvement. Appetite had recovered and weight had stabilized. She was discharged from GI follow-up. No extrapyramidal symptoms, movement disorders, or serious adverse effects were observed at any visit. Neurological examination—performed at each visit by the co-author pediatric neurologist (C.M.) and encompassing all elements assessed by AIMS—was normal throughout. Estimated total metoclopramide exposure under GI supervision was approximately 770 mg.

### 2.3. Case 3—16-Year-Old Female with POTS, ARFID, Hypermobile EDS, and Severe GI Dysmotility

Patient 3 is a 16-year-old female with a complex history encompassing POTS (formally diagnosed by her dysautonomia specialist neurologist), avoidant/restrictive food intake disorder (ARFID, diagnosed with prior hospitalization), hypermobile Ehlers–Danlos syndrome (phenotypically confirmed by a geneticist; genetic testing not performed), abnormal uterine bleeding, anxiety disorder, depression, and adjustment disorder. Her illness trajectory began in spring 2024 with presyncope and orthostatic symptoms while at boarding school, followed by heavy menstrual bleeding, iron deficiency, and an iron infusion that precipitated severe POTS symptoms with significant functional impairment. By next month she developed near-complete GI intolerance characterized by minimal oral intake, intractable nausea, severe postprandial epigastric pain described as ‘stabbing,’ and a weight loss exceeding 20 lbs from her baseline (from 140 to 112 lbs).

Prior to her initial GI visit, she had undergone an extensive inpatient evaluation at a tertiary pediatric center. EGD with biopsies demonstrated normal mucosa throughout the esophagus, stomach, and duodenum. Fluoroscopic upper GI series showed no evidence of superior mesenteric artery syndrome (SMAS). CT angiography of the abdomen demonstrated a narrowed aortomesenteric angle without frank obstruction, and Doppler ultrasound of the celiac and superior mesenteric arteries was normal—collectively excluding SMAS and median arcuate ligament syndrome. Inflammatory markers (ESR, CRP), fecal calprotectin, and celiac disease screening were all negative. Temporary nasogastric tube feeding achieved weight restoration prior to discharge, and she was discharged with diagnoses of ARFID and disorder of gut–brain interaction. Prior pharmacological trials included cyproheptadine (no benefit), lansoprazole, and gabapentin (minimal benefit). Current medications at presentation—all initiated by psychiatry prior to her GI referral—included olanzapine 2.5 mg twice daily (prescribed for appetite and behavioral support related to her ARFID and dysautonomia), fluoxetine 40 mg twice daily, hydroxyzine, gabapentin, famotidine, midodrine 2.5 mg three times daily, ondansetron PRN, and fexofenadine. Olanzapine was continued under psychiatric supervision throughout the metoclopramide course and was subsequently discontinued by psychiatry following her clinical improvement; her sustained GI and weight recovery after olanzapine discontinuation are discussed in Section 3.2.

At her initial GI visit she weighed 52.3 kg—partially recovered from her nadir but with ongoing severe symptoms. She could tolerate only a few bites of food before pain onset, and fluid intake had declined to approximately 40 oz/day. Assessment was consistent with dysautonomia-associated GI dysmotility and functional dyspepsia with visceral hypersensitivity, superimposed on ARFID and significant psychosocial complexity. She met Rome V criteria for functional dyspepsia with overlapping postprandial distress syndrome and epigastric pain syndrome (severe postprandial epigastric pain, intractable nausea, early satiation) [17]. Metoclopramide 5 mg three times daily was initiated. At this weight, the mg/kg/dose was 0.096 mg/kg. The FDA black box warning was explained in detail to the patient and her guardian, including explicit discussion of acute dystonic reactions and tardive dyskinesia. Given the concurrent use of olanzapine—itself a potent dopamine receptor antagonist—the additive D2 receptor blockade was acknowledged, and the family was specifically counseled on enhanced vigilance for any extrapyramidal symptoms, with diphenhydramine prescribed as a rescue agent. Concurrent GI interventions included dietary counseling (small frequent low-fat meals, low-fiber foods, liquid supplements), magnesium glycinate, ginger capsules, hyoscyamine PRN, scopolamine patch (initiated at second visit), diaphragmatic breathing, and structured psychology support through a multidisciplinary team including GI psychology, trauma therapy, and adolescent medicine for ARFID.

At the 2-week follow-up, the patient had gained 2.3 kg, was completing meals, and reported substantially reduced abdominal pain. Nausea remained present but improved. She was admitted to hospital for a planned bowel cleanout and comprehensive motility evaluation, at which time EGD, colonoscopy with biopsies, EndoFLIP, and esophageal manometry were performed—all results were normal, with normal esophageal distensibility on EndoFLIP and no evidence of neuromuscular dysmotility on manometry. Metoclopramide was continued throughout.

At the 3-month visit, she reported nausea occurring only every 1–2 weeks, complete resolution of abdominal pain, stable bowel habits, and significant weight recovery to 61.9 kg (+9.6 kg from nadir at initial visit, now at 68th percentile BMI for age). She was engaged in daily walking, beginning yoga, and had enrolled in formal ARFID treatment (Equip program). Mood was markedly improved. A structured taper of metoclopramide was initiated: 5 mg twice daily, then once daily, then discontinuation over approximately 3 weeks. Total active treatment duration was approximately 10–11 weeks; estimated cumulative dose was approximately 1050 mg. No extrapyramidal symptoms or movement disorders were observed at any point during treatment. Neurological examination—performed at each visit by the co-author pediatric neurologist (C.M.) and encompassing all elements assessed by AIMS—was normal at all visits. At the 9-month follow-up she remained off metoclopramide and had maintained her weight recovery (61.2 kg). She was attending school, was engaged in therapy, and was continuing acupuncture. Residual constipation was addressed with prucalopride.

Table 1 summarizes the clinical characteristics, interventions, and outcomes across all three patients. Table 2 summarizes visit-by-visit metoclopramide dosing, symptom trajectory, and safety outcomes across all three patients.

## 3. Discussion

This case series describes three adolescent females with specialist-confirmed dysautonomia and refractory GI dysmotility who were treated with a standardized short-course, low-dose metoclopramide bridge protocol as part of a multidisciplinary management approach. To our knowledge, this is the first published report specifically describing metoclopramide as a targeted prokinetic bridge strategy in a pediatric dysautonomia population. All three patients demonstrated meaningful symptomatic improvement in nausea and GI function, enabling engagement with dietary, behavioral, and integrative interventions. No extrapyramidal symptoms or serious adverse effects attributable to metoclopramide were observed across the three cases, including in the most complex patient who received concurrent olanzapine—itself a potent D2 receptor antagonist.

### 3.1. Dysautonomia and the Rationale for Prokinetic Bridge Therapy

The GI manifestations of dysautonomia are mechanistically grounded in autonomic dysregulation of gut function. Perturbations in sympathovagal balance can alter gastric emptying, promote visceral hypersensitivity, and impair antroduodenal coordination [6,7]. Among patients with POTS and related orthostatic disorders, studies have shown abnormal gastric emptying in 33–42% of pediatric cohorts, abnormal gastric myoelectrical activity during orthostatic challenge, and evidence of neuropathic dysmotility on antroduodenal manometry [6]. Splanchnic venous pooling and mesenteric hyperemia further contribute to postprandial symptoms by exacerbating relative central hypovolemia [6,7]. Visceral hypersensitivity—mediated through central sensitization and altered gut–brain axis signaling—amplifies symptom perception even in the absence of overt structural dysmotility [7,8].

The standard multidisciplinary approach to dysautonomia-associated GI dysmotility encompasses increased fluid and sodium intake, compression garments, graduated physical reconditioning, dietary modification (small frequent low-fat meals, low-fiber diet), psychological and behavioral therapies, and supplementary integrative interventions [6,7]. While evidence supports the efficacy of this integrated framework, engagement with these strategies is severely impaired when patients are debilitated by continuous nausea, near-total food restriction, and functional collapse. In this context, a time-limited pharmacological intervention capable of providing sufficient GI symptom relief to re-establish oral intake and engagement with the therapeutic program represents a critical clinical bridge.

Metoclopramide’s prokinetic mechanism—accelerating gastric emptying through D2 receptor antagonism and 5-HT4 agonism, and its antiemetic action at the chemoreceptor trigger zone—addresses two of the core symptom drivers in dysautonomia-related GI dysfunction [9,12]. Its rapid onset of action (effect within 1–3 days of initiation) and availability as an inexpensive generic oral preparation make it particularly practical as a bridge agent in this setting. The response observed in all three of our patients—with meaningful GI symptom reduction within 2–6 weeks—is consistent with this mechanistic rationale. We note that in Patient 2, metoclopramide had already produced substantial patient-reported benefit before any GI-directed multidisciplinary intervention was introduced, offering one within-case observation consistent with a drug-specific contribution independent of the broader treatment bundle.

Several alternative prokinetic agents were considered and not selected for these patients. Erythromycin, a motilin agonist, is associated with tachyphylaxis within weeks of initiation and may exacerbate orthostatic symptoms; it is also not approved by the FDA for gastroparesis. Prucalopride, a selective 5-HT4 agonist, is primarily indicated for chronic constipation, with limited published evidence for upper GI prokinetic action in adolescents. Domperidone, a peripheral D2 antagonist with a more favorable central side-effect profile, is not available for routine prescription in the United States and carries cardiac arrhythmogenic concerns. Pyridostigmine, an acetylcholinesterase inhibitor, targets parasympathetic deficiency and is more often used for autonomic indications than for nausea-predominant dysmotility. Within this landscape, metoclopramide—as the only FDA-approved oral prokinetic with combined gastroprokinetic and central antiemetic action—was selected as the most appropriate bridge agent for adolescents with refractory dysautonomia-associated upper GI symptoms.

Patient 3 illustrates a further layer of this pathophysiology: the overlap between POTS, hypermobile Ehlers–Danlos syndrome (hEDS), and disorders of gut–brain interaction. Connective tissue laxity in hEDS is increasingly recognized as a contributor to GI dysmotility and visceral hypersensitivity through mechanisms distinct from, but overlapping with, autonomic dysregulation [18]. In pediatric cohorts, comorbid hypermobility and orthostatic intolerance are associated with significantly worse nausea, disability, and somatization scores than either condition alone, consistent with the clinical severity observed in Patient 3 [19].

### 3.2. Safety Profile and Contextualizing the Black Box Warning

The FDA black box warning issued in 2009 established that metoclopramide can cause tardive dyskinesia (TD), an involuntary movement disorder that may persist after discontinuation, and recommended against use beyond 12 weeks [12]. The warning further states that metoclopramide tablets are ‘not recommended for use in pediatric patients’ due to the risk of TD and other extrapyramidal symptoms (EPSs), as well as methemoglobinemia in neonates [12]. These regulatory recommendations were grounded principally in retrospective case series and small studies predominantly involving elderly adult patients receiving prolonged high-dose treatment. The TD risk estimates of 1–15% now embedded in the older medical literature have been augmented and contextualized by contemporary real-world evidence [13]. We emphasize that this evolving literature does not abrogate the legitimate concerns underlying the FDA warning, particularly with respect to prolonged exposure, elderly patients, and patients with concurrent dopamine receptor blockade.

A landmark 2026 retrospective cohort study, using the IBM MarketScan database of nearly 99 million unique patients over the period 2011–2020, reported a TD incidence of 159.4 per 100,000 person-years (0.37%) among metoclopramide-treated gastroparesis patients—markedly lower than the 1–15% previously cited in national guidelines [13]. Critically, after adjusting for confounding variables, metoclopramide use was not independently associated with increased TD risk among gastroparesis patients (adjusted IRR 1.09; 95% CI 0.92–1.28; *p* = 0.332), suggesting that observed unadjusted differences primarily reflected the underlying risk profiles of patients treated with metoclopramide rather than the drug itself [13]. The number needed to harm was calculated as 2628 person-years of metoclopramide use for one additional TD case. For context, the NNH for extrapyramidal symptoms with olanzapine and risperidone is 10 and 20, respectively [14]. The risk was additionally shown to plateau at cumulative doses of 10,000–15,000 mg—corresponding to approximately 36–54 weeks at full adult doses—well above the estimated cumulative exposures in our three patients (770–1050 mg) [13].

These findings are consistent with the earlier systematic review which estimated the true risk of metoclopramide-induced TD at approximately 0.1% per 1000 patient-years—far below the 1–10% range embedded in regulatory guidance—and identified the foundational studies underpinning the black box warning as methodologically limited by small sample sizes, older patient populations, and prolonged high-dose exposure [12,20].

In the pediatric-specific literature, the systematic review and meta-analysis by Lau Moon Lin et al.—encompassing 108 studies (57 prospective) involving 2699 children aged 18 years or younger—found that the most common adverse effects associated with multiple-dose metoclopramide use in prospective studies were EPS (mean 9%; 95% CI 5–17%), diarrhea (6%), and sedation (6%) [14]. No fatalities were directly attributed to metoclopramide. TD was reported in only two children across the entire reviewed literature, both within case reports rather than prospective studies; symptoms persisted for over 9 and 15 months in those two cases [14]. The EPS reported in pediatric cohorts predominantly represents acute dystonia—a reversible, dose-dependent phenomenon distinct from the chronic, persistent TD that is the primary concern of the black box warning.

A critical distinction must be emphasized: acute EPS (predominantly acute dystonia and akathisia) and TD are mechanistically and clinically separate entities with markedly different risk profiles. Acute dystonia is more common in younger patients, is dose-dependent, appears rapidly (often within the first dose to days), and resolves promptly with drug discontinuation and anticholinergic rescue [12,14]. Of direct relevance to our patient population, the original Bateman et al. epidemiological analysis of 15.9 million UK metoclopramide prescriptions identified the highest dystonia/dyskinesia reporting rate (190.7 reports per million prescriptions) in females aged 12–19 years [21]. This historical finding underscores the importance of explicit family counseling regarding acute dystonic reactions and the provision of diphenhydramine rescue at initiation—both of which were standard elements of our protocol. Tardive dyskinesia, by contrast, is an idiosyncratic complication of prolonged DRBA exposure that develops after months to years of treatment and carries potential for persistence [9,12]. The cumulative doses in our three patients (770–1050 mg over 10–12 weeks) are substantially below any threshold associated with meaningful TD risk in the contemporary literature. Our protocol’s built-in taper—5 mg TID for approximately one month, BID for one month, then QD for one month—further minimizes cumulative exposure and avoids abrupt discontinuation.

The combination of metoclopramide and olanzapine in Patient 3 warrants specific comment. Olanzapine had been initiated by psychiatry prior to metoclopramide, not concurrently, and was continued under psychiatric supervision for the duration of the metoclopramide course. Both agents are dopamine receptor-blocking agents, and their concurrent use theoretically increases the risk of EPS through additive D2 receptor blockade [12,13]. This combination was acknowledged explicitly at metoclopramide initiation, with enhanced counseling regarding EPS recognition and diphenhydramine rescue prescribed. No extrapyramidal symptoms were observed over 10–11 weeks of concurrent use, consistent with findings that antipsychotic co-prescription increases relative TD risk while absolute risk remains low at short treatment durations [9,13]. Olanzapine was subsequently discontinued by psychiatry following her clinical improvement; her weight recovery and GI symptom improvement were sustained after this discontinuation, which argues against attribution of the GI improvement to olanzapine alone. We nonetheless emphasize that olanzapine introduces an important confounder for both appetite-related and safety observations in this case, and we recommend that clinicians exercise heightened vigilance and employ structured EPS monitoring—including formal AIMS assessment at baseline and at each follow-up visit—when metoclopramide is co-prescribed with antipsychotic agents.

### 3.3. The Bridge Therapy Framework and Multidisciplinary Integration

We utilized metoclopramide strictly as a time-limited bridge—not a maintenance therapy. We define “bridge therapy” operationally as a pharmacological intervention with four explicit components: (a) a clearly defined start aligned with refractory symptoms preventing engagement with sustainable care, (b) a pre-specified taper protocol, (c) a defined therapeutic endpoint (in our cases, engagement with multidisciplinary rehabilitative care), and (d) discontinuation upon endpoint achievement. This operational framework builds on prior work describing time-limited, symptom-targeted interventions to enable engagement with definitive multidisciplinary care in children with otherwise intractable gastroparesis [22]. In each case, the drug was initiated with a planned taper and discontinuation endpoint, and was embedded within a broader multidisciplinary care framework including dietary modification, neuromodulators, integrative interventions (magnesium glycinate, ginger, Iberogast, acupuncture), behavioral and psychological support, and autonomic-directed therapies (midodrine, compression garments, hydration, salt loading). This framing aligns with current expert recommendations for the management of co-occurring dysautonomia and disorders of gut–brain interaction, which emphasize individualized, symptom-targeted, biopsychosocial care and the use of pharmacotherapy as an adjunct to—not a replacement for—lifestyle and behavioral interventions [6,7]. Tricyclic antidepressants such as amitriptyline have demonstrated efficacy in pediatric functional GI disorders and were used as neuromodulators in this series [23].

It is important to emphasize that the symptomatic improvement observed in all three patients cannot be attributed to metoclopramide in isolation. Each patient received a comprehensive multidisciplinary intervention that included dietary modification, neuromodulators, laxatives, integrative therapies, psychological support, and autonomic-directed treatment. The contribution of metoclopramide should be understood as one component of a multifactorial therapeutic response, and this case series cannot disentangle the relative contributions of individual interventions. The clinical observation that the protocol enabled engagement with the broader multidisciplinary framework—rather than producing symptomatic improvement in isolation—is the central rationale for the bridge therapy concept presented here.

The concept of bridge therapy is particularly important in this population because the therapeutic strategies with the strongest evidence for long-term benefit in dysautonomia-associated GI dysmotility—graded physical reconditioning, dietary rehabilitation, cognitive-behavioral therapy, ARFID-specific interventions—all require a baseline level of oral intake and functional engagement that is simply unavailable when patients are experiencing continuous nausea and near-complete food avoidance. In Patient 3, the most complex case, 10–11 weeks of metoclopramide bridging achieved a weight recovery of nearly 10 kg from nadir and enabled engagement with the ARFID program, GI psychology, trauma therapy, and structured physical activity—a trajectory that was not achievable in the preceding 4 months of conservative management at a tertiary center, including a temporary feeding tube. A comparable multidisciplinary bridge strategy—combining pelvic floor physiotherapy, occupational therapy, psychological support, and motility-directed medication—has been reported to avert surgical feeding tube placement in an adolescent with hEDS-related feeding difficulty and comorbid POTS, supporting the generalizability of this approach [24].

Consensus from the Pediatric Assembly of the American Autonomic Society identifies gastrointestinal symptoms—particularly nausea—as a core comorbid domain in pediatric autonomic dysfunction, recommending the Nausea Severity Scale as the validated screening instrument for this population [7]. The absence of standardized outcome measurement is a recognized limitation of our series, and future prospective work in this area should incorporate validated instruments including the Nausea Severity Scale and ROME V criteria.

### 3.4. Proposed Safety Monitoring Protocol

Based on the safety experience of this series and the supporting literature, we propose the following monitoring framework for clinicians considering short-course metoclopramide in adolescents with dysautonomia-associated GI dysmotility:

Prior to initiation: Confirm dysautonomia diagnosis by specialist evaluation. Review concomitant medications for additional D2 receptor-blocking agents (antipsychotics, prochlorperazine, promethazine). Calculate weight-based dose (target ≤ 0.15 mg/kg/dose; maximum 5 mg per dose in adolescents). Assess for absolute contraindications (prior TD or dystonic reaction to metoclopramide, Parkinson’s disease, epilepsy, pheochromocytoma, GI hemorrhage or obstruction, significant hepatic or renal impairment). Counsel patient and family regarding black box warning, signs and symptoms of acute dystonia (involuntary neck or facial movements, tongue protrusion, oculogyric crisis) and akathisia (motor restlessness), instruction to stop the medication immediately if these occur, and to administer diphenhydramine 1 mg/kg (maximum 50 mg) orally or intramuscularly. Prescribe diphenhydramine as a rescue agent at initiation. Document verbal informed consent including black box warning discussion.

During treatment: Assess for EPS at each follow-up visit by direct neurological examination (gait, involuntary movements, facial and orolingual movements). When co-prescribing with antipsychotic agents, consider formal AIMS assessment at baseline and at each visit. Monitor weight, dietary intake, and functional status at each encounter. Planned taper should be discussed at initiation and reassessed at each visit. Duration should not exceed 12 weeks without explicit re-evaluation of the risk–benefit profile.

At discontinuation: Confirm absence of EPS or movement disorder at final visit. Document neurological examination findings. Instruct patient to return if GI symptoms recur acutely, and to contact the prescribing clinician if any abnormal movements develop after discontinuation.

**Figure 1:**
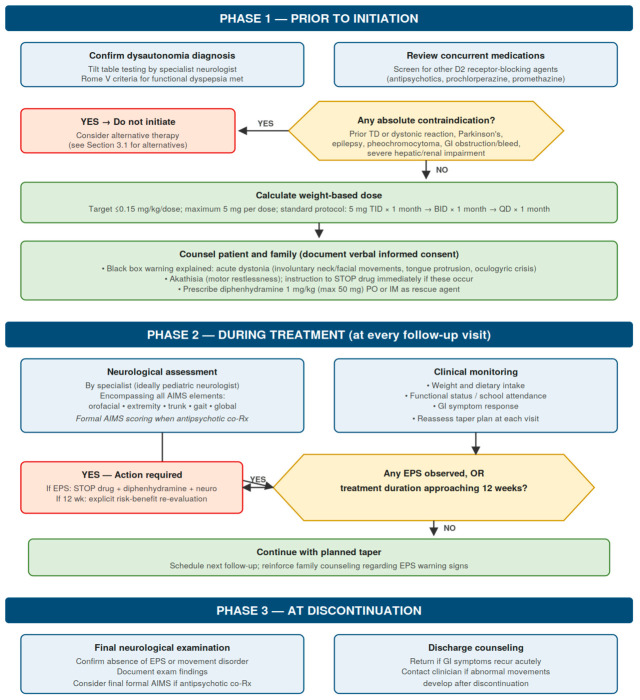
Proposed safety monitoring algorithm for short-course metoclopramide use in adolescents with dysautonomia-associated GI dysmotility. The algorithm depicts three phases—prior to initiation, during treatment, and at discontinuation—with explicit decision points for dose calculation, family counseling, diphenhydramine rescue prescription, AIMS-equivalent neurological assessment at each visit (with formal AIMS scoring recommended when co-prescribing with antipsychotic agents), and the 12-week duration ceiling beyond which explicit risk–benefit re-evaluation is required.

The monitoring framework described above is summarized as an algorithm in Figure 1.

## 4. Limitations

Several limitations of this case series must be acknowledged. The sample size of three patients precludes generalizability and does not permit statistical inference; in particular, a three-patient series cannot independently evaluate the incidence of rare adverse events such as tardive dyskinesia. All patients are adolescent females, limiting applicability to other demographic groups. Outcomes were assessed by clinical interview and physician assessment; no validated symptom instruments (such as the Nausea Severity Scale or Gastroparesis Cardinal Symptom Index) were employed, precluding quantitative comparison of symptom severity across visits. Formal gastric emptying scintigraphy was not performed in any patient. The diagnosis of “dysautonomia-associated GI dysmotility” in our cases therefore represents a clinical working diagnosis based on specialist-confirmed dysautonomia, fulfillment of Rome V criteria for functional dyspepsia, exclusion of structural and inflammatory disease, and clinical response to prokinetic therapy—rather than radiologically confirmed gastroparesis. Each patient received extensive concurrent multidisciplinary interventions; consequently, the observed symptomatic improvement should be considered multifactorial and cannot be attributed to metoclopramide in isolation. The safety observation of no adverse effects is encouraging but based on a small convenience sample. Although neurological assessment was performed at each visit by a board-certified pediatric neurologist (C.M.) encompassing all clinical elements of the AIMS, the formal AIMS scoring instrument was not applied. The concurrent administration of olanzapine in Patient 3 introduces an additional confounder for both safety and outcome interpretation in that case. Finally, this series represents the experience of a single institution and prescribing clinician, introducing potential selection bias.

## 5. Conclusions

This case series presents three adolescent females with specialist-confirmed dysautonomia and refractory GI dysmotility who were treated with a structured, short-course, low-dose metoclopramide bridge protocol. To our knowledge, following a structured PubMed search, no prior case series has specifically reported this strategy in pediatric dysautonomia. All three patients achieved meaningful GI symptom improvement that enabled engagement with broader multidisciplinary and integrative care; this improvement was multifactorial and cannot be attributed to metoclopramide in isolation. No extrapyramidal symptoms or serious adverse effects were observed. Estimated cumulative metoclopramide exposures of 770–1050 mg across all three cases are below the threshold (10,000–15,000 mg) at which TD risk has been shown to plateau in contemporary real-world pharmacoepidemiological data [13]. These observations are hypothesis-generating and should not be interpreted as redefining the established safety profile of metoclopramide in pediatric populations; a three-patient series cannot independently evaluate rare adverse events. They do, however, support the need for prospective investigation of carefully monitored, weight-dosed, time-limited use in this specific clinical context.

Prospective studies in this population are urgently needed. Such studies should incorporate as essential—rather than optional—components: validated symptom instruments including the Nausea Severity Scale, the Rome V Pediatric Diagnostic Questionnaire [17], the Gastroparesis Cardinal Symptom Index, the Functional Disability Inventory, and PROMIS pediatric measures for quality of life; standardized autonomic diagnostic criteria; objective EPS monitoring using the formal AIMS instrument; formal gastric emptying assessment; and patient-reported functional outcomes. Such data would provide the evidence base necessary to inform updated evidence-based guidelines for this underserved and therapeutically challenging population.

## Figures and Tables

**Table 1:** Summary of clinical characteristics, dysautonomia diagnosis, metoclopramide protocol, and outcomes in three adolescents with dysautonomia-associated gastrointestinal dysmotility.

Characteristic	Patient 1	Patient 2	Patient 3
Age/Sex	13F	16F	16F
Dysautonomia Diagnosis	OH with reactive tachycardia (TTT-confirmed by neurologist)	Orthostatic hypotension (TTT-confirmed by neurologist)	POTS (TTT-confirmed by neurologist, dx 10/2024)
Key Comorbidities	Anxiety	Anxiety, ADHD	ARFID, hEDS, anxiety, depression, AUB
GI Symptom Duration	3–4 years	2–3 years	Active exacerbation ~4 months; prior ARFID 2 years
Rome V FD Diagnosis	FD-PDS subtype	FD overlapping PDS + EPS; functional constipation	FD overlapping PDS + EPS
Presenting GI Symptoms	Daily nausea, intermittent vomiting, early satiety, constipation	Chronic nausea, postprandial fullness, early satiety, constipation, episodic lower abdominal pain	Severe postprandial epigastric pain, intractable nausea, bloating, severely restricted intake, constipation, >20 lb weight loss
Prior GI Workup	EGD (normal); fluoroscopic UGI (normal); abdominal/brain imaging (normal); labs (normal)	Labs comprehensive (normal); EKG/echo (normal); no endoscopy indicated	EGD × 2 (normal); UGI series (normal); CTA abdomen (no obstruction); Doppler US (normal); labs including calprotectin (normal); colonoscopy + EndoFLIP + manometry (all normal)
Baseline Weight/mg/kg/dose	51.9 kg/0.096 mg/kg	56.6 kg/0.088 mg/kg	52.3 kg/0.096 mg/kg
Prior Treatments Failed	Cyproheptadine, Iberogast, ginger, amitriptyline 10 mg	Supportive (nausea limiting intake); metoclopramide started by prior provider	Cyproheptadine, lansoprazole, gabapentin, nasogastric tube feeding (temporary)
Notable Co-medications	Amitriptyline, midodrine, oral contraceptive	Escitalopram, methylphenidate, PPI	Olanzapine 2.5→5 mg BID, fluoxetine, midodrine, gabapentin, ondansetron PRN
Metoclopramide Protocol	5 mg TID → 5 mg BID (wk 8) → self-tapered off	5 mg TID (formalized from prior) → 5 mg PRN (self-reduced) → stopped at ~11 wk	5 mg TID → 5 mg BID → 5 mg QD → discontinued over 3 wks (total ~10–11 wk)
Estimated Cumulative Dose	~900–1000 mg	~770 mg	~1050 mg
Adverse Effects	None	None	None (no EPS despite concurrent olanzapine)
GI Outcome	Complete resolution of nausea/vomiting; normalized bowel habits; dietary variety expanded	Marked improvement in nausea; constipation improved; PPI successfully weaned; appetite recovered	Dramatic improvement: nausea episodes 1–2×/week at 3 months; pain resolved; +9.6 kg weight recovery; engaged in ARFID therapy and school
Neurological Exam at Final Visit	Normal; no movement disorder	Normal; no movement disorder	Normal; no movement disorder
Disposition	Discharged from GI; follow-up PRN	Discharged from GI; prucalopride for constipation	Continued multidisciplinary follow-up; off metoclopramide at 9 months

Abbreviations: OH, orthostatic hypotension; POTS, postural orthostatic tachycardia syndrome; TTT, tilt table test; ARFID, avoidant/restrictive food intake disorder; hEDS, hypermobile Ehlers–Danlos syndrome; AUB, abnormal uterine bleeding; EGD, esophagogastroduodenoscopy; UGI, upper gastrointestinal; EPS, extrapyramidal symptoms; TID, three times daily; BID, twice daily; QD, once daily; PRN, as needed; PPI, proton pump inhibitor.

**Table 2:** Visit-by-visit metoclopramide dose, symptom status, and safety observations for each patient.

Visit	Approximate Timing	Patient 1— Dose/Status	Patient 2— Dose/Status	Patient 3— Dose/Status
1	Initiation	5 mg TID started. Daily nausea, vomiting, early satiety, constipation. Black box warning, diphenhydramine rescue counseled.	5 mg TID formalized (started 6 wk prior by outside provider). Nausea, constipation, postprandial fullness. Black box warning re-counseled.	5 mg TID started. Severe postprandial pain, intractable nausea, restricted intake (40 oz fluids/day). Black box warning, diphenhydramine rescue counseled. Olanzapine co-rx noted; enhanced EPS monitoring discussed.
2	2–6 weeks	5 mg TID continued. Nausea improved; eating better; BMs 4–5×/wk. Wt +1.4 kg.	5 mg TID continued. Still nauseous but improved. Constipation partially improved. Weight −1.4 kg (nausea-related decreased intake).	5 mg TID continued. Wt +2.3 kg. Completing meals. Abdominal pain markedly reduced. Nausea persisting. Hospitalization for cleanout + motility studies (all normal).
3	8–11 weeks	Weaned to 5 mg BID. No nausea, no pain. Regular BMs. Dietary variety improving. Neurological exam normal.	Self-reduced to 5 mg BID. ‘Doing a lot better from nausea standpoint.’ Constipation improved. No pain. Wants fewer meds.	(3 months) 5 mg TID → started wean to BID. Nausea 1–2×/wk. Pain resolved. Wt 61.9 kg (+9.6 kg from nadir). School planning. Neurological exam normal.
4	~12 wks/ Follow-up	Self-tapered off. Complete resolution. Stable off prokinetics. Discharged.	Stopped (~11 wk GI-supervised). Improved appetite, weight recovered. No EPS. Discharged. Prucalopride for constipation.	(9 months) Off metoclopramide. Weight 61.2 kg (maintained). Attending school. ARFID therapy ongoing. No EPS at any visit.

Abbreviations: TID, three times daily; BID, twice daily; BMs, bowel movements; EPS, extrapyramidal symptoms; Wt, weight.

## Data Availability

The original contributions presented in this study are included in the article. Further inquiries can be directed to the corresponding author.

## Abbreviations

The following abbreviations are used in this manuscript:

ANS	Autonomic nervous system
AIMS	Abnormal Involuntary Movement Scale
ARFID	Avoidant/restrictive food intake disorder
AUB	Abnormal uterine bleeding
BID	Twice daily
DRBA	Dopamine receptor-blocking agent
EGD	Esophagogastroduodenoscopy
EPS	Extrapyramidal symptoms
FD	Functional dyspepsia
FD-EPS	Epigastric pain syndrome (FD subtype)
GI	Gastrointestinal
hEDS	Hypermobile Ehlers–Danlos syndrome
OH	Orthostatic hypotension
PDS	Postprandial distress syndrome
POTS	Postural orthostatic tachycardia syndrome
PPI	Proton pump inhibitor
PRN	As needed
QD	Once daily
TD	Tardive dyskinesia
TID	Three times daily
TTT	Tilt table test
UGI	Upper gastrointestinal
